# etymologia:** Candida**

**DOI:** 10.3201/eid1402.070236

**Published:** 2008-02

[kan′-di-də], from the Latin—*candidus* (glowing white)

A genus of yeastlike Fungi Imperfecti (for which no sexual reproductive stage is known) of the family *Cryptococcaceae* that produce yeast cells, mycelia, pseudomycelia, and blastospores. When grown in the laboratory, *Candida* appears as large, round, white or cream colonies on agar plates. *C. albicans* infection (or thrush) features distinctive white mouth lesions; “albicans” means becoming white. *C. dubliniensis, *first identified in 1995 at the University of Dublin, is an opportunistic pathogen that can cause both superficial and invasive infections, particularly in the immunocompromised.

